# Feasibility and Toxicity of Interval-Compressed Chemotherapy in Asian Children and Young Adults with Sarcoma

**DOI:** 10.3390/jpm13040668

**Published:** 2023-04-14

**Authors:** Jia-Hui Huang, Shu-Huey Chen, Yu-Mei Liao, Yu-Chien Kao, Wan-Ling Ho, Hsi Chang, Min-Lan Tsai, Hsin-Lun Lee, Chia-Chun Kuo, Sung-Hui Tseng, Chia-Yau Chang, Kevin Li-Chun Hsieh, Long-Sheng Lu, Yin-Ju Chen, Jeng-Fong Chiou, Tsung-Han Hsieh, Yun-Ru Liu, Wayne Hsu, Wei-Tang Li, Yu-Chung Wu, Wei-Ciao Wu, Jinn-Li Wang, Jia-Jia Tsai, Keita Terashima, Chikako Kiyotani, Tai-Tong Wong, James S. Miser, Yen-Lin Liu

**Affiliations:** 1 Department of Pediatrics, Taipei Medical University Hospital, Taipei 110, Taiwan; 2 Department of Pediatrics, School of Medicine, College of Medicine, Taipei Medical University, Taipei 110, Taiwan; 3 Taipei Cancer Center, Taipei Medical University, Taipei 110, Taiwan; 4 Departments of Pediatrics, Shuang Ho Hospital, Ministry of Health and Welfare, Taipei Medical University, Zhonghe, New Taipei 235, Taiwan; 5 Division of Hematology and Oncology, Department of Pediatrics, Kaohsiung Medical University Hospital, Kaohsiung 807, Taiwan; 6 Department of Pathology, School of Medicine, College of Medicine, Taipei Medical University, Taipei 110, Taiwan; 7 Department of Pathology, Taipei Medical University Hospital, Taipei 110, Taiwan; 8 Department of Radiology, School of Medicine, College of Medicine, Taipei Medical University, Taipei 110, Taiwan; 9 Department of Radiation Oncology, Taipei Medical University Hospital, Taipei 110, Taiwan; 10 Department of Health Care Administration, College of Management, Taipei Medical University, Taipei 110, Taiwan; 11 Ph.D. Program for Cancer Molecular Biology and Drug Discovery, College of Medical Science and Technology, Taipei Medical University and Academia Sinica, Taipei 110, Taiwan; 12 Department of Radiation Oncology, Wan Fang Hospital, Taipei Medical University, Taipei 111, Taiwan; 13 Department of Physical Medicine and Rehabilitation, School of Medicine, College of Medicine, Taipei Medical University, Taipei 110, Taiwan; 14 Department of Physical Medicine and Rehabilitation, Taipei Medical University Hospital, Taipei 110, Taiwan; 15 Neuroscience Research Center, Taipei Medical University Hospital, Taipei 110, Taiwan; 16 Department of Medical Imaging, Taipei Medical University Hospital, Taipei 110, Taiwan; 17 TMU Research Center of Cancer Translational Medicine, Taipei Medical University, Taipei 110, Taiwan; 18 Graduate Institute of Biomedical Materials and Tissue Engineering, College of Biomedical Engineering, Taipei Medical University, Taipei 110, Taiwan; 19 International PhD Program in Biomedical Engineering, Taipei Medical University, Taipei 110, Taiwan; 20 Department of Medical Research, Taipei Medical University Hospital, Taipei 110, Taiwan; 21 Center for Cell Therapy, Taipei Medical University Hospital, Taipei Medical University, Taipei 110, Taiwan; 22 International PhD Program for Cell Therapy and Regeneration, Taipei Medical University, Taipei 110, Taiwan; 23 Joint Biobank, Office of Human Research, Taipei Medical University, Taipei 110, Taiwan; 24 Division of General Surgery, Department of Surgery, Taipei Medical University Hospital, Taipei 110, Taiwan; 25 Division of Plastic Surgery, Department of Surgery, Taipei Medical University Hospital, Taipei 110, Taiwan; 26 Division of Thoracic Surgery, Department of Surgery, Taipei Medical University Hospital, Taipei 110, Taiwan; 27 Division of Thoracic Surgery, Department of Surgery, Shuang Ho Hospital, Ministry of Health and Welfare, Taipei Medical University, Zhonghe, New Taipei 235, Taiwan; 28 Department of Pediatrics, Wan Fang Hospital, Taipei Medical University, Taipei 111, Taiwan; 29 Children’s Cancer Center, National Center for Child Health and Development, Tokyo 157-8535, Japan; 30 Graduate Institute of Clinical Medicine, College of Medicine, Taipei Medical University, Taipei 110, Taiwan; 31 Division of Pediatric Neurosurgery, Department of Neurosurgery, Taipei Medical University Hospital, Taipei 110, Taiwan; 32 Taipei Neuroscience Institute, Taipei Medical University, Taipei 110, Taiwan; 33 Department of Pediatrics, City of Hope National Medical Center, Duarte, CA 91010, USA

**Keywords:** round cell sarcoma, interval-compressed chemotherapy, children, adolescent and young adult, VDC/IE, granulocyte colony-stimulating factor

## Abstract

**Simple Summary:**

Interval-compressed chemotherapy was evaluated in Asian children and young adults with sarcomas to assess the feasibility of this treatment strategy. Ultimately, the goal is to improve survival in this group of individuals with high-risk malignancies without increasing short-term or long-term toxicities. The feasibility of this strategy in Asian patients with sarcomas needed to be established prior to evaluating the efficacy of this approach in a prospective trial.

**Abstract:**

Twelve Asian patients with sarcoma received interval-compressed (ic-) chemotherapy scheduled every 14 days with a regimen of vincristine (2 mg/m^2^), doxorubicin (75 mg/m^2^), and cyclophosphamide (1200–2200 mg/m^2^) (VDC) alternating with a regimen of ifosfamide (9000 mg/m^2^) and etoposide (500 mg/m^2^) (IE), with filgrastim (5–10 mcg/kg/day) between cycles. Carboplatin (800 mg/m^2^) was added for CIC-rearranged sarcoma. The patients were treated with 129 cycles of ic-VDC/IE with a median interval of 19 days (interquartile range [IQR], 15–24 days. Median nadirs (IQR) were neutrophil count, 134 (30–396) × 10^6^/L at day 11 (10–12), recovery by day 15 (14–17) and platelet count, 35 (23–83) × 10^9^/L at day 11 (10–13), recovery by day 17 (14–21). Fever and bacteremia were observed in 36% and 8% of cycles, respectively. The diagnoses were Ewing sarcoma (6), rhabdomyosarcoma (3), myoepithelial carcinoma (1), malignant peripheral nerve sheath tumor (1), and *CIC-DUX4* Sarcoma (1). Seven of the nine patients with measurable tumors responded (one CR and six PR). Interval-compressed chemotherapy is feasible in the treatment of Asian children and young adults with sarcomas.

## 1. Introduction

The outcome of children and young adults with sarcoma has improved dramatically with the use of systemic chemotherapy in the context of multimodality treatment [1]. In spite of these improvements, there is still a need for better therapy for the sarcomas. The modern chemotherapy regimens for high-risk sarcomas incorporate non-cross-resistant chemotherapy regimens that are largely derived from the development of chemotherapy in Ewing sarcoma (ES). In a randomized controlled trial, the addition of ifosfamide and etoposide (IE) to standard chemotherapy with doxorubicin, vincristine, cyclophosphamide (VDC), and dactinomycin significantly improved the survival of patients with localized ES [2]. 

Based on these results of alternating chemotherapy, a strategy of increasing the dose intensity has been evaluated in ES in two ways. Increasing the dose of the agents did not improve the outcome [3]; however, giving interval-compressed (ic-) chemotherapy with VDC/IE scheduled every 14 days with the use of granulocyte colony stimulating factor (G-CSF) was found to improve the event-free survival of localized ES [4] compared to VDC/IE scheduled every 21 days. The concept of ic-VDC/IE has also been successfully incorporated in the treatment of intermediate-risk and high-risk rhabdomyosarcoma [5,6]. The other prospective treatments for sarcomas in children and young adults that need to be evaluated in the future include immunotherapy [7] and targeted therapy [8]. 

Currently, there is a lack of data for the use of ic-chemotherapy in Asian children and young adults. This study demonstrates the feasibility and toxicity of interval-compressed chemotherapy in treating sarcomas in children and young adults in Asia. 

## 2. Materials and Methods

### 2.1. Patient Eligibility 

From December 2016 to December 2020, Asian patients younger than 30 years of age with newly diagnosed sarcomas treated with ic-VDC/IE at two university healthcare systems in Taiwan were included in this retrospective analysis of the feasibility, toxicity, and early efficacy of this regimen. 

### 2.2. Diagnosis and Staging

The diagnosis of sarcoma was established morphologically and with the use of immunohistochemistry and the detection of gene rearrangements to determine the specific diagnosis when indicted. The molecular diagnosis of fusion genes was performed by fluorescent in situ hybridization (FISH) or ribonucleic acid sequencing (RNA-Seq) (see below). 

Staging work-up included computerized tomography (CT) or magnetic resonance imaging (MRI) of the primary site, CT of the lungs, and whole-body bone scan. The TNM Staging System (American Joint Committee on Cancer, 8th Edition Staging System for Soft Tissue Sarcoma of the Extremities or Trunk) was used [9]. 

### 2.3. Fluorescence In Situ Hybridization (FISH)

To detect fusion genes and confirm their fusion candidates, we performed FISH for *EWSR1*, *FLI1*, *FOXO1*, *CIC*, and *DUX4* break-apart as modified from previously described methods [10]. Commercial probes for *EWSR1* and *FLI1* were purchased from ZytoVision (Bremerhaven, Germany). Custom probes were made by bacterial artificial chromosomes (BAC) clones flanking the genes of interest according to the UCSC genome browser (http://genome.ucsc.edu, accessed on 20 July 2019) and obtained from BACPAC sources of Children’s Hospital of Oakland Research Institute (Oakland, CA, USA; http://bacpac.chori.org, accessed on 20 July 209). DNA from each BAC was isolated according to the manufacturer’s instructions. The BAC clones were labeled with fluorochromes (fluorescent-labeled dUTPs, Enzo Life Sciences, Farmingdale, NY, USA) by nick translation and validated on normal metaphase chromosomes. The 4 μm-thick FFPE slides were deparaffinized, pretreated, and hybridized with denatured probes. After overnight incubation, the slides were washed, stained with 4′,6-diamidino-2-phenylindole (DAPI), mounted with an antifade solution, and then examined on an Olympus BX63 automated fluorescence microscope (Evident corporation, Tokyo, Japan) controlled by GenASIs software, Version 8.0.1 (Applied Spectral Imaging, Carlsband, CA, USA). 

### 2.4. RNA Sequencing

RNA sequencing was performed with TruSeq RNA Exome (Illumina, San Diego, CA, USA) that converted total RNA extracted from formalin-fixed paraffin-embedded (FFPE) tissues into template molecules of known strand origin, followed by sequence-specific capture of coding RNA. Paired-end RNA-seq at read lengths of 150 base pairs were performed with the HiSeq 2000 (Illumina). After being independently aligned by STAR (version 2.3) against the human reference genome (hg19), the reads were analyzed by STAR-Fusion algorithm for fusion discovery. 

### 2.5. Treatment

Patients were treated with interval-compressed chemotherapy regimens with vincristine (2 mg/m^2^/dose; maximum, 2 mg/dose), doxorubicin (75 mg/m^2^/cycle), and cyclophosphamide (1200–2200 mg/m^2^/cycle) (VDC) alternating with Ifosfamide (9000 mg/m^2^/cycle) and Etoposide (500 mg/m^2^/cycle) (IE). Carboplatin (800 mg/m^2^/cycle) was added with IE for the *CIC*-rearranged sarcoma [11]. The interval-compressed chemotherapy cycles (ic-VDC/IE) were scheduled every 14 days; therapy was delivered when the neutrophil count had recovered to ≥750 × 10^6^/L, and the platelet count had recovered to ≥75 × 10^9^/L without transfusions. 

To support the patients through neutrophil nadirs and to compress treatment intervals, granulocyte colony-stimulating factor (G-CSF; filgrastim 5–10 mcg/kg/day) was given subcutaneously daily, starting from 24 to 36 h after the last dose of chemotherapy to the recovery of neutrophil count to ≥750 × 10^6^/L. Neutrophil and platelet counts were evaluated 3 times a week following each cycle of chemotherapy until recovery.

Treatment of the primary site with surgery was performed before chemotherapy in patients who required urgent decompression or if upfront resection was considered feasible. Otherwise, treatment of the primary site with surgery, radiation, or both was planned to begin at week 10–15 after initiation of systemic chemotherapy for patients with nonmetastatic disease or after maximal systemic control for patients with metastatic disease.

### 2.6. Evaluation of Treatment Response and Adverse Events

Treatment response was assessed by two-dimensional (2D) measurements according to the World Health Organization [12], which defined complete response (CR) as 100% reduction of tumor size, partial response (PR) as 50–99% reduction of tumor size, stable disease (SD) as 0–49% reduction or 0–24% enlargement of tumor size, and progressive disease (PD) as ≥25% enlargement of tumor size. 

The adverse events were evaluated by the National Cancer Institute Common Terminology Criteria for Adverse Events (NCI CTCAE) version 5.0. Acute adverse events of a cycle were defined as complications that occurred during the administration of chemotherapy or before the next chemotherapy cycle began. Significant adverse events included infections, shock, mucositis, cytopenias, impaired liver function, and abnormal renal function. 

### 2.7. Statistical Analysis

Clinical characteristics of patients, including age at diagnosis, gender, tumor size, stage, treatments, and chemotherapy regimen were collected. Toxicities were collected for each chemotherapy cycle. The interval between chemotherapy cycles was defined by the number of days between the first day of two consecutive cycles; for the last cycle, recovery was defined by the number of days until neutrophil and platelet counts returned to ≥750 × 10^6^/L and ≥75 × 10^9^/L without transfusions, respectively. Continuous variables were presented by median and interquartile ranges (IQRs) and compared by the Kruskal-Wallis test. Progression-free survival (PFS) and overall survival (OS) were calculated by the Kaplan-Meier method and compared by the log-rank test. 

## 3. Results

### 3.1. Patient Characteristics

From December 2016 to December 2020, twelve patients from Taiwan, Japan, or China with newly diagnosed sarcoma were treated at our institutions with ic-VDC/IE and were eligible for analysis. There were 6 males and 6 females with a median age of 14 years (range, 3–29 years) at diagnosis. Their diagnoses included six ES, three alveolar rhabdomyosarcoma, one myoepithelial carcinoma, one malignant peripheral nerve sheath tumor (MPNST), and one *CIC-DUX4* sarcoma. The primary tumors originated in the soft tissue (*n* = 9) or bone (*n* = 3). The most common primary sites were trunk (*n* = 10), extremities (*n* = 1), and retroperitoneum (*n* = 1). Six of the twelve patients had metastatic diseases; the most common metastatic site was bone (*n* = 4). The clinical characteristics of the patients are shown in Table 1. 

### 3.2. Intensification of Chemotherapy Intervals

The ic-VDC/IE treatment cycles were planned at 14-day intervals. A total of 129 cycles of chemotherapy were given at a median interval of 19 days (interquartile range [IQR], 15–24 days). The intervals between neoadjuvant chemotherapy cycles (*n* = 64; median [IQR] = 16 [14–19] days) were significantly shorter than the intervals between concurrent chemoradiotherapy cycles (*n* = 15; median [IQR] = 21 [18–26] days) and the intervals after local control (*n* = 50, median [IQR] = 23 [20–32] days) (*p* = 0.0001). 

### 3.3. Toxicity and Recovery 

The toxicities during chemotherapy are shown in Table 2. Grade 3 and 4 hematological toxicities were common: neutropenia (92%), thrombocytopenia (62%), and anemia (33%). The most common grade 3 and 4 non-hematological toxicities were fever (36%), urinary tract infection (8%), bacteremia (6%), and mucositis (3%). No patient developed septic shock or infection-associated death during ic-VDC/IE. 

**Figure 1:**
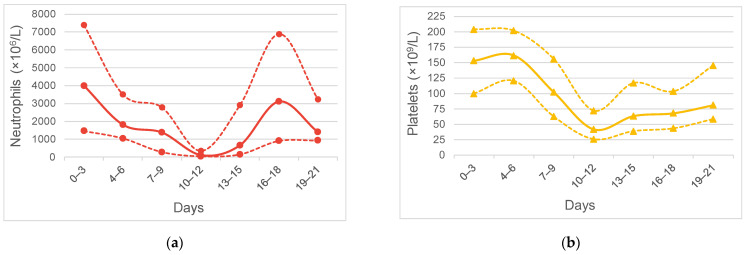
Hematological toxicity of 129 cycles of ic-VDC/IE in young Asian patients: (**a**) Neutrophil count of cycles; (**b**) Platelet count of cycles. Solid lines are median counts, and dashed lines are 25th% and 75th% counts, respectively.

Patients had their neutrophil count decreased to a median nadir (IQR) of 134 (30–396) × 10^6^/L at median (IQR) day 11 (10–12) that had recovered by day 15 (14–17) with the use of G-CSF (Figure 1a). After the cessation of G-CSF use, the neutrophil count dropped back to the near-normal range. Patients had their platelet count decreased to a median nadir (IQR) of 35 (23–83) × 10^9^/L at median (IQR) day 11 (10–13) that recovered by day 17 (14–21) (Figure 1b). The recovery of the platelet count was slower than the neutrophil count; no patients in this cohort used thrombopoietin. 

### 3.4. Treatment Response and Outcomes

Initial response to neoadjuvant chemotherapy alone was evaluable in the nine patients with measurable disease; responses included one CR, six PR, one SD, and one PD (overall response rate = 78%). Among the six patients with PR after ic-VDC/IE neoadjuvant chemotherapy, four achieved CR after local control with surgery and radiotherapy (*n* = 2) or radiotherapy alone (*n* = 2). The non-responders were the patient with *CIC*-rearranged sarcoma stage IV (No.9; SD) and the patient with MPNST (No.5; PD). The three other patients had primary tumor resection prior to chemotherapy. 

All four patients with Ewing sarcoma without metastases at diagnosis achieved long-term systemic control and remain in CR. One patient (No.4) developed second malignancy with acute lymphoblastic leukemia at 17 months after an initial diagnosis of localized ES and remains in CR of both diseases. The other 2 patients with non-metastatic disease at diagnosis, MPNST (No.5) and ARMS (No.6), had disease progression at 11 and 31 months after diagnosis, respectively. 

Five of six patients with metastatic disease had initial response to ic-VDC/IE, including 1 CR and 4 PR; however, all six patients with metastatic sarcoma progressed at 9–37 months after initial diagnosis, regardless of best response to treatment. 

At a median follow-up of 43 months, all patients with localized sarcoma who received ic-VDC/IE remained in remission. The 2-year PFS in patients with localized disease at diagnosis was 83%; the 2-year PFS in patients with metastases at diagnosis was 33% (*p* = 0.05). The 2-year OS in patients with localized disease at diagnosis was 83%; the 2-year OS in patients with metastatic disease at diagnosis was 67% (*p* = 0.28). 

The treatment and outcomes of patients were summarized in Table 3. 

## 4. Discussion

Sarcomas are a heterogenous group of malignant tumors arising in bone, soft tissue, and the peripheral nervous system. Most sarcomas in children and young adults are high-grade. Molecular diagnostics are now utilized in combination with histopathology and immunohistochemistry to characterize sarcomas. The most common round cell sarcomas in this age group, characterized by relatively small, round-to-oval, undifferentiated cells, include Ewing sarcoma/primitive neuroectodermal tumor (ES/PNET) and rhabdomyosarcoma [13]. In children and young adults, sarcomas have a slight male predilection, with overlapping but different epidemiological features and clinical presentations across histological and molecular types [14]. 

Race/ethnicity has an impact on the incidence and treatment outcomes of sarcomas in children and young adults [15,16]. In ES, the incidence in Caucasians is 2-fold higher than that in Asian/Pacific Islanders and 9-fold higher than that in African Americans [16]. Extraskeletal ES accounts for approximately 20% of all ES with a better prognosis in most studies [17,18]. Intriguingly, non-Caucasian patients had a higher incidence of Extraskeletal ES compared to Caucasians (36 vs. 19%) and an inferior survival [18,19]. In ARMS, the incidence rate in Caucasians is 4-fold higher than that in African Americans and 8-fold higher than that in other races [20]. These observations suggest the importance of careful study of the incidence and longitudinal follow-up of patients of different races and ethnic groups with sarcomas. 

The standard chemotherapy regimens for treating sarcoma have evolved over the last five decades. The first multiagent chemotherapy regimen for ES was a combination of vincristine, dactinomycin, and cyclophosphamide (VAC) [21]. This was based on phase two trials of each drug that showed activity in the disease [22,23]. This combination improved the outcome for Ewing Sarcoma at the time but only modestly. When doxorubicin was approved for patients, phase two data showed activity in Ewing Sarcoma [24]. This agent was then added to the standard regimen at the time, VAC, with improvement in outcome for patients with both nonmetastatic [25] and metastatic disease [26]. In the 1980s, ifosfamide was shown to have activity in a phase two trial in recurrent patients [27]. The combination of etoposide with ifosfamide in the treatment of Ewing sarcoma showed significantly increased activity suggesting that this combination might be non-cross-resistant with the standard regimen of VAC plus doxorubicin [28]. This led to an intergroup study in the United States that demonstrated the benefit of adding this new regimen to the standard regimen [2]. 

Similar to the experience with Ewing Sarcoma, the standard regimen of VAC was developed based on phase two data for each agent alone and was shown to have benefit in newly diagnosed rhabdomyosarcoma [29]. Subsequent studies demonstrated that doxorubicin had activity in rhabdomyosarcoma and significantly benefitted patients with alveolar rhabdomyosarcoma [30]. The new combination of ifosfamide plus etoposide combined with vincristine was evaluated in patients with stage III rhabdomyosarcoma and was demonstrated to have activity that was equivalent to the standard regimen of VAC [31,32]. The use of ic-VDC/IE in rhabdomyosarcoma has been evaluated in initial studies and requires further investigations in prospective, randomized trials [5,6]. The treatment of Ewing-like sarcomas is less well established because of their rarity; however, the current treatment regimens for these entities are based on the treatment of Ewing Sarcoma. 

The use of a non-cross-resistant regimen in our patients is based on the Ewing sarcoma trial in the United States which added the combination of ifosfamide and etoposide to the standard regimen of VAC plus doxorubicin with 5-year overall survival (5y-OS) of 72% [2]. The addition of IE to VDC in a non-cross-resistant chemotherapy regimen also improved survival in non-metastatic ES in Japan (5-year overall survival, 80.1%) [17] and in Taiwan (5-year overall survival, 61.6%) [33]. 

The concept of dose intensity related to outcome was initially evaluated in breast cancer [34,35]. It was also evaluated in sarcomas demonstrating the importance of dose intensity in this population [36]. In these studies, increasing dose intensity was related to an improved outcome. 

Following the development of G-CSF, interval compression of chemotherapy was developed to intensify the delivered dosing [37]. The feasibility of ic-VDC/IE was first demonstrated in a pilot study in the United States of children with ES/PNET, rhabdomyosarcoma, and other advanced soft tissue sarcomas. The median interval of chemotherapy cycles was 16 days, representing a 1.27-fold increase of intensity comparing with the traditional schedule of 21-day intervals [37]. 

Further trials in ES and rhabdomyosarcoma have confirmed the usefulness of ic-VDC/IE by increasing the dose intensity without increasing the total dose [4,5]. A large cooperative group trial evaluating interval compression of this chemotherapy regimen demonstrated a significant improvement in the outcome for patients with nonmetastatic ES. The efficacy of ic-VDC/IE has been validated in adult ES studies [38,39] and is being evaluated in a large European trial with promising early data [40,41]. By contrast, the use of dose escalation of VDC/IE has not improved the event-free survival and overall survival of localized ES [3,42]. Furthermore, a cohort study of 81 adult Chinese patients with ES receiving every-3-week VDC/IE found that chemotherapy delay of more than 3 days was associated with worse survival by multivariate Cox regression analysis, highlighting the importance of interval intensity [43].

Toxicities associated with ic-VDC/IE chemotherapy include febrile neutropenia, non-neutropenia related infection, anemia, thrombocytopenia, and mucositis. In our study, we did not observe grade 3 and 4 renal toxicity or encephalopathy. According to the report of Weigel et al., the most common toxicity was infection with and without neutropenia in 50 and 60% of patients during ic-VDC/IE, rates similar to those in our cohort [5]. As bone marrow toxicity and infections were the major toxicities observed in ic-VDC/IE, dose reductions instead of dose delays should be considered when these side effects occur [44]. 

The major clinical benefits of interval-compressed chemotherapy are: (1) to enable rapid shrinkage of the primary tumor that can enhance the success rate of surgery at the primary site after maximal response has been achieved; and (2) to prevent the development of metastatic disease. Further interval-intensive systemic control can be continued during the period of local therapy [45]. Thus, the main pro of interval-compressed chemotherapy is the improvement of survival in nonmetastatic sarcoma as confirmed in the two large trials in ES in the U.S. and Europe [4,41]. In addition, the ability to complete chemotherapy in a shorter period of time is appealing to many patients. Further, to give more cycles of interval-compressed chemotherapy prior to surgery may enhance local control. The major con of interval-compressed chemotherapy is the loss of rest time between chemotherapy cycles; however, the toxicity for the courses was not more than conventional chemotherapy [39].

Metastatic disease [20,46] is the major challenge in treating children and young adults with sarcomas despite the good results of ic-VDC/IE in localized sarcomas. Patients with metastatic sarcomas often have a good initial response to chemotherapy; however, this is usually followed by progression within 2 years. In metastatic ES/PNET, the addition of IE [47], dose escalation of VDC/IE [48], or the use of ic-VDC/IE [4] have not been associated with improved EFS or OS. In high-risk rhabdomyosarcomas, ic-VDC/IE was associated with better EFS only in embryonal rhabdomyosarcoma but not in ARMS [5]. In our study, three of the three patients with ARMS had disease progression in contrast to two progressions out of the six patients with ES. More aggressive treatment is required to improve the treatment outcome of metastatic sarcomas. To further intensify the systemic therapy in sarcomas, European studies utilized HDC/ASCR with busulfan/melphalan that improved the EFS and OS in localized ES [49] and achieved a similar outcome of whole-lung irradiation in ES with pulmonary metastases [50]. In a single institutional study in the U.S., adding topotecan to busulfan/melphalan as the HDC/ASCR regimen achieved a 10-year OS of 78% in patients at 1st CR and of 66% in patients at 2nd CR or 1st PR of metastatic or relapsed ES [51]. In rhabdomyosarcoma, the use of thiotepa and melphalan in Japan has resulted in a 3-year PFS > 50% for ARMS [20,52]. 

The survival of patients with *CIC*-rearranged sarcoma is significantly worse than patients with conventional ES [53]. The 5-year OS of less than 50% in both localized and metastatic disease demonstrates the need for better therapy for this histology. VDC/IE can induce durable complete responses, however [54,55]. 

New approaches to treat metastatic sarcomas include targeted agents and immunotherapy. A novel targeted agent, ganitumab, a monoclonal antibody against IGF-1R, is being tested in a phase III clinical trial (NCT02306161). Further, tyrosine kinase inhibitors and anti-angiogenic agents are being evaluated in clinical trials [8]. Novel immunotherapy with HER2 chimeric antigen receptor T cells has been tested in an early-phase clinical trial. The efficacy of immune checkpoint inhibition has been limited in ES and other sarcomas [56,57,58]. Further, most sarcomas are immune “cold” tumors; only alveolar soft part sarcoma and undifferentiated pleomorphic sarcoma have responded to immune checkpoint inhibitors [8]. Recently, down-regulation or deletion of major histocompatibility complex class I antigens was found to be a common phenomenon in sarcomas that restricts tumor-specific CD8^+^ T cell response [7]. This may explain the failure of immune checkpoint inhibition in the treatment of many sarcomas. 

Our study has several limitations. First, it is a retrospective analysis of a small number of cases. Given the rarity of sarcomas, however, our data demonstrates the feasibility of ic-VDC/IE in Asian patients. The clinical benefit of interval-compressed chemotherapy should be thoroughly studied in histology-driven treatment protocols and validated in future prospective studies. Second, the median interval between cycles in our study was 19 days, which was longer than the 15–16 days reported in Western literature, which might, in part, be the result of delayed platelet recovery. Whether the use of thrombopoietin receptor agonists can further compress the interval between chemotherapy cycles by maintaining platelet counts may be worth exploring [59]. Third, we did not observe long-term disease control in all patients with metastatic sarcomas although four of them achieved transient CR or PR. This observation highlights the need for more improved therapy, such as high-dose chemotherapy with autologous stem cell rescue, for patients with metastatic sarcomas. 

## 5. Conclusions

In this study, we demonstrate that interval-compressed chemotherapy is feasible in treating Asian children and young adults with sarcoma with tolerable toxicity. Neoadjuvant ic-VDC/IE may enhance local control with rapid shrinkage of primary tumors and addresses the systemic disease early in the treatment. New approaches to the treatment of metastatic disease are needed and may include more aggressive regimens, targeted therapy, immunotherapy, or high-dose consolidation chemotherapy. Our experience needs to be validated in prospective, histology-driven studies, especially in rhabdomyosarcoma, in a larger population of Asian patients by incorporating ic-VDC/IE into multidisciplinary approaches that integrate surgery, radiotherapy, and chemotherapy.

## Figures and Tables

**Table 1:** Clinical characteristics, histopathology, and molecular features of Asian patients with sarcomas.

No.	Age	Sex	Histology	Fusion Gene (Method)	Initial Presentation	Primary Site	1° Tumor Diameter	Metastases	Stage	Initial Surgery
1	4	F	Ewing	*EWSR1* (FISH)	Upper back pain × 5 wk	T4 spinous process; T3–T6 epidura	2.4 cm	–	II	R1 resection
2	16	M	Ewing	*EWSR1-FLI1* (FISH)	Palpable mass × 1 yr	Supraclavicular, right	3.3 cm	–	II	R0 resection *
3	3	F	Ewing	*EWSR1*; *FLI1* (FISH)	Paraparesis × 2 d	T2 spinous process; T2–T4 epidura	3.5 cm	–	II	R1 resection
4	15	M	Ewing	*EWSR1* (FISH)	Low back pain × 4 wk	L3–L4 lamina & soft tissue, left	3.7 cm	–	II	R2 resection
5	13	M	MPNST	–	Leg pain × 2 wk	L2 paravertebral soft tissue	6.6 cm	–	III	R2 resection
6	15	F	ARMS	*PAX3-FOXO1* (FISH)	Buttock pain × 4 wk	Perianal soft tissue	9.6 cm	–	III	Bx & Colonoscopy
7	29	M	Ewing	*EWSR1*; *FLI1* (FISH)	Abdominal fullness × 4 wk	Retroperitoneum	14 cm	Mesentery	IV	Needle Bx
8	14	F	ARMS	*FOXO1* (FISH)	Acute urinary retention	Perineum, left	7.1 cm	BM, bones (multiple)	IV	Cystoscopic Bx
9	10	F	URCS	*CIC-DUX4* (10q) (FISH)	Leg pain × 6 wk	Pleura, left	14 cm	Bone (left femur)	IV	R0 resection *
10	8	M	MEC ^#^	*EWSR1-ETV1* (RNA-Seq)	Low back pain × 4 wk	Forearm, right	4.3 cm	BM, bones (multiple)	IV	R1 resection
11	17	M	Ewing	*EWSR1*; *FLI1* (FISH)	Chest pain & dyspnea × 2 wk	Pleura, left	26.5 cm	Lung (left)	IV	Needle Bx
12	14	F	ARMS	*PAX3-FOXO1* (FISH)	Cough × 1 wk	Chest wall, left	10.6 cm	Bones	IV	Needle Bx

Abbreviations: BM, bone marrow; Bx, biopsy; FISH, fluorescent in situ hybridization; PR, partial response; R0, no residual microscopic disease; R1, microscopic residual disease; R2, gross residual disease; RNA-Seq, ribonucleic acid sequencing; RT, radiotherapy; URCS, undifferentiated round cell sarcoma; wk, week(s); yr, year(s). * The surgical margin was free but close (<1 mm). ^#^ The tumor histology showed a malignant round-to-spindle cell neoplasm, most consistent with a high-grade malignancy with overlapping features of myoepithelial carcinoma and Ewing Sarcoma.

**Table 2:** Grade 3 and 4 toxicities observed during 129 cycles of ic-VDC/IE chemotherapy (number of cycles with the toxicities and their percentage of total cycles are presented).

Grade	3		4	
	*n*	%	*n*	%
Neutropenia *	21	16%	98	76%
Thrombocytopenia *	42	33%	37	29%
Anemia *	42	33%	0	0%
Fever	0	0%	46	36%
Urinary tract infection	10	8%	0	0%
Bacteremia	0	0%	8	6%
Shock	0	0%	0	0%
AST	2	2%	0	0%
ALT	1	1%	0	0%
Bilirubin	1	1%	0	0%
Creatinine	0	0%	0	0%
Mucositis	4	3%	1	1%
Anorexia	0	0%	0%	0%
Nausea	0	0%	0%	0%
Vomiting	0	0%	0%	0%

* Definitions of hematological toxicities: Neutropenia grade 3, 500–999 × 10^6^/L; grade 4, <500 × 10^6^/L. Anemia grade 3, hemoglobin < 8 g/dL or transfusion-indicated; grade 4, life-threatening consequences or urgent intervention needed. Thrombocytopenia grade 3, 25–49 × 10^9^/L; grade 4, <25 × 10^9^/L.

**Table 3:** Treatment and outcomes of Asian patients with sarcomas.

No.	Age	Sex	Dx	Stage	Initial Op	Chemo Res.	2nd Op	RT Dose (cGy)	Tx Duration *	Best Response	Prog-Ression	Status	Survival
1	4	F	Ewing	II	R1	–	–	4000	8.8 m	CR	–	NED	4 y 4 m
2	16	M	Ewing	II	R0 ^#^	–	–	5040	8.9 m	CR	–	NED	4 y 3 m
3	3	F	Ewing	II	R1	–	–	4320 (Spine)	7.5 m	CR	–	NED	2 y 8 m
4	15	M	Ewing	II	R2	PR	–	4500 (Spine); 5500 (Boost)	8.0 m	CR	–	SMN	2 y 5 m
5	13	M	MPNST	III	R2	PD	R2	6600 (Spine) ^#^; 7800 (Boost) ^#^	11.5 m	PD	11 m	DOD	1 y 6 m
6	15	F	ARMS	III	Bx	PR	R1	5040	12.2 m	CR ^†^	2 y 7 m ^†^	AWD	3 y 6 m
7	29	M	Ewing	IV	Bx	PR	R0 ^§^	5000	11.3 m	CR	1 y 11 m	DOD	2 y 10 m
8	14	F	ARMS	IV	Bx	PR	R2	4500	12.1 m	PR	12 m	DOD	1 y 6 m
9	10	F	*CIC*	IV	R0 ^#^	SD	–	5000	9.9 m	SD	1 y	DOD	1 y 4 m
10	8	M	MEC	IV	R1	PR	–	3600 (spine); 4500 (forearm)	8.7 m	CR ^†^	3 y 1 m ^†^	NED	3 y 9 m
11	17	M	Ewing	IV	Bx	PR	R2	4500 (pleura); 5040 (tumor)	9.0 m	PR	1 y 2 m	AWD	2 y 11 m
12	14	F	ARMS	IV	Bx	CR	R0 ^‡^	3600 (chest wall); 4140 (breast)	13.6 m	CR	2 y 7 m	AWD	3 y 7 m

Abbreviations: AWD, alive with disease; Bx, biopsy; Chemo Res., response to initial interval-compressed chemotherapy; DOD, died of disease; CR, complete response; NED, no evidence of disease; Op, surgery; PD, progressive disease; PR, partial response; R0, tumor resection with no residual microscopic disease; R1, tumor resection with microscopic residual disease; R2, tumor resection with gross residual disease; RT, radiotherapy; SD, stable disease; SMN, second malignant neoplasm (acute lymphoblastic leukemia at 1 year and 5 months after diagnosis); Tx, treatment; y/m, years/months. * Treatment duration: The time interval from initial diagnosis to the end of the first-line treatment protocol. ^#^ The dosing unit was centigray equivalent by proton beam therapy. ^§^ The surgical margin was free but close (<1 mm). ^†^ High-dose chemotherapy with autologous stem cell rescue has been performed. ^‡^ Resection of residual lesion revealed fibrosis without evidence of malignancy.

## Data Availability

The data presented in this study are available on request from the corresponding authors. The data are not publicly available due to privacy considerations.

## Abbreviations

ARMS	alveolar rhabdomyosarcoma;
G-CSF	Granulocyte colony-stimulating factor;
ic-	Interval compressed;
IE	Ifosfamide, etoposide;
IQR	interquartile range;
MEC	myoepithelial carcinoma;
MPNST	malignant peripheral nerve sheath tumor;
PNET	primitive neuroectodermal tumor;
UTI	urinary tract infection;
VDC	Vincristine, doxorubicin, cyclophosphamide
